# Complex interactions in soil-transmitted helminth co-infections from a cross-sectional study in Sri Lanka

**DOI:** 10.1093/trstmh/try068

**Published:** 2018-07-25

**Authors:** Hannah C Lepper, Joaquín M Prada, Emma L Davis, Sharmini A Gunawardena, T Déirdre Hollingsworth

**Affiliations:** 1 Zeeman Institute for Systems Biology and Infectious Disease Epidemiology Research, Mathematics Institute and School of Life Sciences, University of Warwick, UK; 2 School of Veterinary Medicine, University of Surrey, Guilford, UK; 3 Faculty of Medicine, University of Colombo, Colombo, Sri Lanka; 4 Big Data Institute, Li Ka Shing Centre for Health Information and Discovery, Nuffield Department of Medicine, University of Oxford, Oxford, UK

**Keywords:** *Ascaris lumbricoides*, co-infection, epidemiology, *Necator americanus*, Sri Lanka, *Trichuris trichiura*

## Abstract

**Background:**

Co-infection with multiple soil-transmitted helminth (STH) species is common in communities with a high STH prevalence. The life histories of STH species share important characteristics, particularly in the gut, and there is the potential for interaction, but evidence on whether interactions may be facilitating or antagonistic are limited.

**Methods:**

Data from a pretreatment cross-sectional survey of STH egg deposition in a tea plantation community in Sri Lanka were analysed to evaluate patterns of co-infection and changes in egg deposition.

**Results:**

There were positive associations between *Trichuris trichiura* (whipworm) and both *Necator americanus* (hookworm) and *Ascaris lumbricoides* (roundworm), but *N. americanus* and *Ascaris* were not associated. *N. americanus* and *Ascaris* infections had lower egg depositions when they were in single infections than when they were co-infecting. There was no clear evidence of a similar effect of co-infection in *Trichuris* egg deposition.

**Conclusions:**

Associations in prevalence and egg deposition in STH species may vary, possibly indicating that effects of co-infection are species dependent. We suggest that between-species interactions that differ by species could explain these results, but further research in different populations is needed to support this theory.

## Introduction

Soil-transmitted helminths (STHs) are widespread, with 1.45 billion people infected globally in 2010^1^ and more than 830 million children in need of preventive treatment in 2016.^2^ Often, all three major STHs (*Ascaris lumbricoides* [roundworm]; *Trichuris trichiura* [whipworm]; *Necator americanus* and *Ancylostoma duodenale* [hookworm]) will be present in a community^3–5^ and co-infections with more than one STH are common.^6^ For example, in one community in Brazil, 24.1% of the population were infected with both hookworm and *Ascaris*, which was a higher prevalence than single infections of either.^7^

Given the high prevalence of STH co-infections, it is important to investigate the effects and drivers of co-infection and the consequences for STH epidemiology and morbidity.^7,8^ Correlated exposures and host predisposition could lead to positive associations between species and increased intensity of infection. *Ascaris*, *Trichuris* and *Ancylostoma duodenale* share faecal–oral transmission routes, and exposure factors such as sanitation and toilet facilities are expected to correlate with co-infection.^6,9^ Within-host interactions between species have also been studied, as helminths have many opportunities for interaction within the host via resource sharing and host-immune responses.^7–11^ However, little is known about the nature of these interactions in humans and there have been few epidemiological studies addressing them.

Epidemiological studies of all three STHs in human populations are common, and some have studied co-infection. One study of a rural community in Brazil found a positive association in the prevalence of *N. americanus a*nd *Ascaris* and that egg deposition was higher in dual infections,^7^ although egg deposition must be interpreted with care, as it is affected by both the number of adult worms and the fecundity of individual worms. A study of mass drug administration in Burundi found that infections with hookworm, *Ascaris* and *Trichuris* were all associated with greater odds of multiple infection, with the strongest effect in *Trichuris*.^12^ A large review study that collated STH data from 44 different epidemiological studies and analysed the independence of worm species prevalences found positive associations between species were common, especially between *A. lumbricoides* and *T. trichiura*, although they did not assess hookworm infections as separate *A. duodenale* and *N. americanus*.^6^ Interestingly, this study also found that there was heterogeneity in associations by geographic regions.^6^

Previous studies have investigated how co-infections of more than one helminth species may be cooperative or antagonistic and found that interactions between species are highly complex.^9,13^ It has been shown that co-infections of intestinal nematodes in rodent models under laboratory conditions are competitive, with reduced or suboptimal establishment of one of the co-infecting species.^9^ In the same study, however, the results of analysis of helminth infections in wild rodent populations did not corroborate this finding, suggesting that being infected with one intestinal helminth increased the likelihood of infection with another.^9^

STHs use immunoregulation to escape host immunity in the gut, and it has been theorized that the immunoregulation of the different STH species may be synergistic within the host, resulting in a less hostile immune environment in co-infections.^7,8,10,11^ Studies of the immunology of co-infection in humans and animal models have provided some support for this theory. One study found more down-regulation of the specific, pro-inflammatory T helper 1 (Th1) immune response in *N. americanus*, *A. lumbricoides* and *Schistosoma mansoni* co-infections compared with single infections, although infection intensity was not affected by co-infection.^10^ Another study by Geiger et al.^14^ compared low- and high-intensity single infections and co-infections of *A. lumbricoides* and *T. trichiura* and found that both co-infection and heavy-intensity infections had reduced parasite-specific immune responses and Th1 responses, suggesting the importance of worm burden rather than co-infection effects, although this could also be an indication of host immune capacity–related predisposition.

Here, we build on these previous studies by analysing historical data on all STH species present in two tea plantations in Sri Lanka (*A. lumbricoides, N. americanus* and *T. trichiura*),^5,15^ focusing on potential associations in prevalence and egg deposition between the different worm species. First, we compared the odds of infection of each species in the presence of co-infections. Second, we assessed egg deposition for each worm species in single infections and co-infections. These analyses provide information about both the impact of co-infection for epidemiology at the population level and infection outcome at the individual level. There are few studies that combine both individual and population-level measures of effect of STH co-infection in human populations, but it is potentially informative about the mechanism and outcomes of within-host interactions.

## Materials and methods

### Study population

A detailed description of the data collection and study population can be found in previous publications.^16–18^ Briefly, the study population was two tea plantation communities in Sri Lanka, Ayr in the Western province and Maliboda in the Sabaragamuwa province. There were a total of 477 participants (155 in Ayr and 322 in Maliboda). The two sites were broadly similar, with the main occupation of estate labourer in both plantations (24.8% in Maliboda and 27.1% in Ayr). Maliboda had a younger age profile than Ayr (2–50 y [median 11 y] and 2–76 y [median 18 y], respectively). The distribution of income was higher in Ayr (median 2000 Sri Lankan rupees [SLR]) compared with Maliboda (median 1500 SLR), and Ayr had a higher proportion of the population with access to toilet facilities (75.5% compared with 37.3%). The 5 to 18-year-olds had been treated once a year with mebendazole for 5 y prior to data collection, but they had not been treated for 10–11 months before this study took place.

### Data collection

Data were collected after written consent was obtained from participants in April 2000. A single stool sample was taken alongside a questionnaire of relevant demographic information (including age, occupation, education level) and exposure information (hygiene and sanitation, toilet facilities).^16–18^ Stool samples were analysed using the Kato–Katz technique on one slide.^19^ Although different hookworm species cannot be distinguished by egg morphology, it was assumed that all hookworm eggs were *N. americanus*, as it has been found that this is the only species that causes human infection in Sri Lanka.^15^ All participants were treated with mebendazole after sampling.

The data used here are from the preliminary pretreatment survey, which was followed up with repeated monthly resampling from the same community over the next year.

### Statistical analysis

Statistical analysis was carried out using R version 3.4.1 (R Project for Statistical Computing, Vienna, Austria) and the packages ggplot2, lmtest, and MASS.^20–23^

#### Associations between worm species

We used multiple logistic regression to assess associations between worm species and calculate odds ratios (ORs) of infection with each species given infection with co-infecting species. We considered the additional factors of age, plantation and water source in the models. These variables were chosen to avoid multicollinearity and to reflect variables that were highlighted as important factors in the original studies.^16,18^ Plantation correlated strongly with access to toilet facilities, so only plantation was used as a binary categorical variable, with Ayr and Maliboda as values. Age was included as a categorical variable, with the categories 2–13, 14–18, 19–30 and >31 y. These categories were chosen to match the categories in the original studies^16,18^ and similar studies.^7^ Water source was divided into four categories: water from waterfalls, pipe-borne water, river water and well water.

We used a stepwise method to build the regression models and test for effect modification, adding one factor at a time to the models. For each species model we first assessed the evidence for effect modification between the two species by including it in the model (e.g., *Ascaris ~N. americanus*×*Trichuris*). If the effect modification term was significant, we maintained the interaction in the next model steps and if it was not, the species were included as separate factors (e.g., *Ascaris ~N. americanus*+*Trichuris*). For each additional variable added, the following steps were followed:

1. We assessed effect modification from the additional variable in the ORs of the variables of interest, the co-infecting species, using either:
  - For multilevel variables age and water source, a likelihood ratio (LR) test comparing the model with the effect modification to the model with the factors included separately or
  - For the binary variable plantation, by looking at the significance of the effect modification term. If the LR test or the effect modification term was significant, the factor was maintained as an effect modifier in the next step, otherwise the factors were included separately.
2. We assessed the importance of the variable in explaining variation in the outcome of interest, the odds of infection with the model species, using an LR test comparing the model chosen in the previous step with a model not including the factor at all. If this test was significant, the factor was included in the final model.

This procedure aimed to produce the model best supported by the data available. We also used McFadden’s R^2^ to provide an additional measure of the amount of variation in the data explained by the chosen model.^24^

#### Egg deposition between infection type groups

We used negative binomial generalized linear models (GLMs) to test for an effect of presence of other STH infections on egg deposition. Negative binomial models were used because egg deposition is overdispersed. Egg deposition models were built the same way as the logistic regression models, adding age, plantation and water source using a stepwise method and LR tests. For each species, one model was built to directly test the hypothesis that egg depositions in the presence of any co-infection was different from single infections, and a second model tested the egg deposition in the presence of each other species separately. Raw egg counts were used in all analyses. The reported counts in the text and figures are transformed to eggs per gram of stool (EPG) for clarity. We used a variance-based R^2^, described for GLMs in Zhang,^25^ to provide an approximate measure of the variation in the data explained by each model.

## Results

### Associations between worm species

**Figure 1.**
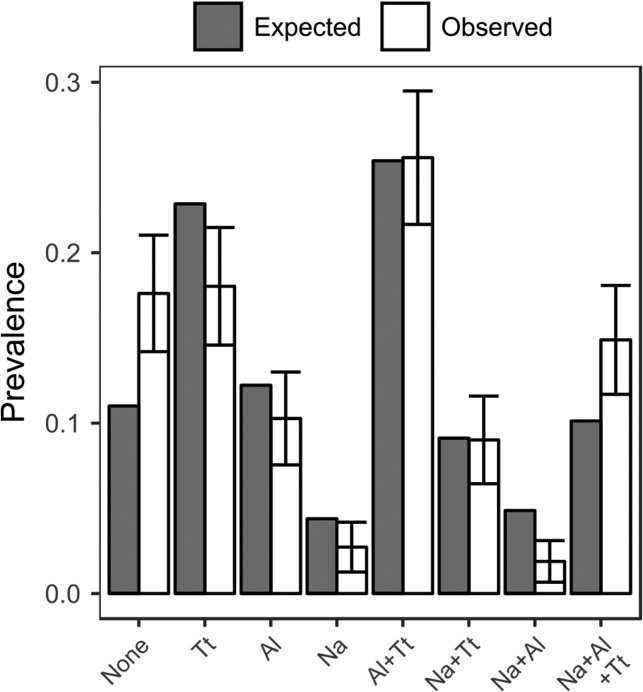
Observed and expected prevalences of single infections and co-infections. Observed values were calculated as the number of cases in an infection type group divided by the total number of participants. Expected values were calculated using three-way contingency tables. Bars on observed values are 95% confidence intervals. Na: *N. americanus*; Al: *A. lumbricoides*; Tt: *T. trichiura.*

Overall there was a 28.9% (136/477), 52.6% (251/477) and 67.5% (322/477) prevalence of *N. americanus*, *Ascaris* and *Trichuris* infections, respectively. The prevalences of single and co-infections are presented in Figure 1. Co-infection was more common overall than single infection, with 245 (51.4%) individuals co-infected with at least two parasite species and 148 (31.0%) singly infected. In the population, 51.2% were 2–13 y, 9.0% were 14–18 y, 13% were 19–30 y and 26.8% were ≥31 y old. A total of 32.5% of the population lived in Ayr and 67.5% in Maliboda.

There were positive associations between *Trichuris* and both other species but no association between *N. americanus* and *Ascaris*. Results of this statistical analysis, including ORs, are presented in Table 1. Figure 1 shows the observed proportions of each type of infection and the proportions that would be expected if the species were distributed randomly with respect to each other in the population. The proportion of individuals uninfected and triply co-infected is greater than would be predicted by chance, while single- and dual-infection proportions are mostly smaller than expected. No effect modification between species was observed to be supported by the data in any model. There was a positive association between the 19–30 y age group and *Ascaris*, but no association was found between age and *Trichuris* or *N. americanus*, and neither plantation nor water source were associated with infection by any species. McFadden’s R^2^ suggests that in each model <1% of the variation in the data is explained by co-infecting species.

**Table 1.** Logistic regressions assessing associations between species, with all model variables, ORs and McFadden’s R^2^s for each model. Species variables are the presence or absence of infection

Species	Variable	OR (95% CI)	p-Value	McFadden’s R^2^ (%)
*Ascaris*	*N. americanus*	1.1 (0.7 to 1.8)	NS	5.1
	*Trichuris*	2.5 (1.6 to 3.7)	<<0.0001	
	<13 y	1	–	
	13–18 y	1.5 (0.8 to 3.0)	NS	
	19–30 y	2.7 (1.5 to 5.1)	<0.005	
	≥30 y	1.1 (0.7 to 1.8)	NS	
*N. americanus*	*Ascaris*	1.2 (0.8 to 1.8)	NS	4.5
	*Trichuris*	3.2 (1.9 to 5.5)	<<0.0001	
*Trichuris*	*Ascaris*	2.4 (1.6 to 3.6)	<<0.0001	7.3
	*N. americanus*	3.2 (1.9 to 5.5)	<<0.0001	

CI: confidence interval; NS: not significant, p>0.05.

### Egg deposition

**Figure 2.**
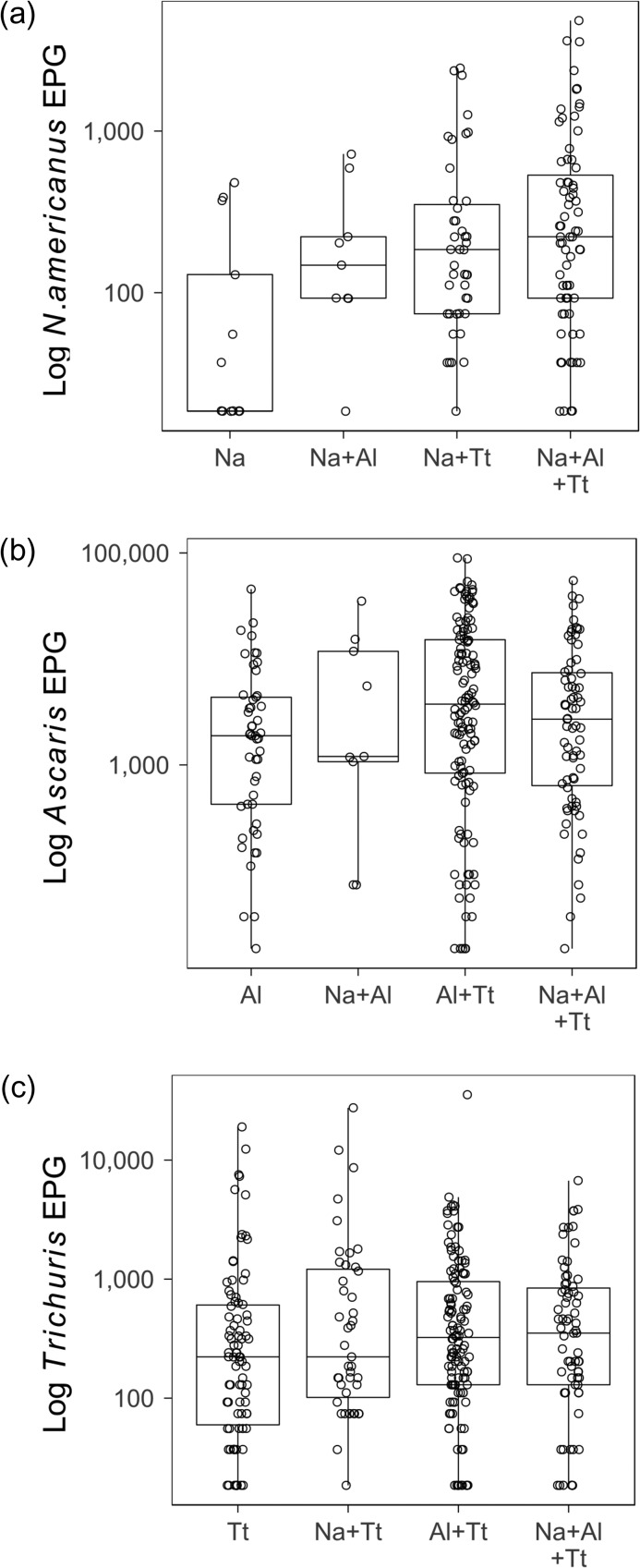
Egg deposition distributions by infection type group. Egg deposition was measured as EPG. Medians, interquartile ranges and individual data points are plotted on a log scale. (a) The egg deposition from *N. americanus* infections, including single infections, infections with *Ascaris* or *Trichuris* and triple infections. (b) The egg deposition from *Ascaris* infections. (c) The egg deposition from *Trichuris* depositions. Na: *N. americanus*; Al: *A. lumbricoides*; Tt: *T. trichiura.*

Overall, among those infected (with a positive egg count), median egg deposition in EPG was 185.0 (interquartile range [IQR] 74.0–450.3) for *N. americanus*, 2701.0 (IQR 675.2–10 878.0) for *Ascaris* and 277.5 (IQR 111.0–809.4) for *Trichuris* (Figure 2). Distributions of egg depositions in each co-infection type are presented in Figure 2, using a log scale to reduce overdispersion.

The results of analysis of the effect of co-infection in egg depositions indicated that both *Ascaris* and *N. americanus* had slightly higher egg counts (Table 2) in co-infections compared with single infections. For *Trichuris* egg deposition, the presence of a co-infection was not a significant factor, although there were significant results for effect modification from age and plantation. Only plantation had a significant effect, with decreased deposition in Maliboda (Table 2). Results from the models that included co-infecting species presence separately are presented in Table 3. Egg depositions in *Ascaris* and *N. americanus* were slightly higher when *Trichuris* was also present (Table 3). The model for *Trichuris* was more complex. There was a significant effect of the presence of *N. americanus*, increasing deposition slightly, after controlling for plantation and water source. Plantation and water source improved the likelihood of the model despite neither of these factors having a detectable effect on their own. There was a significant effect modification of *N. americanus* from plantation. The two *Trichuris* models differed, with different factors included, suggesting neither model is reliable and not supporting an effect of co-infection on *Trichuris* egg deposition.

**Table 2.** Negative binomial generalized linear regression assessing the relationship between egg deposition and the presence of any co-infection, with all model variables, regression coefficients and a variance-based R^2^

Species	Variable	Coefficient (eggs/54 mg) (95% CI)	p-Value	Variance-based R^2^ (%)
*Ascaris*	Any co-infection	0.8 (0.3 to 1.2)	<0.001	0.8
*N. americanus*	Any co-infection	1.4 (0.6 to 2.0)	<0.0005	0.5
*Trichuris*	Any co-infection	−0.1 (−1.0 to 0.8)	NS	0.3
	<13 y	0	–	
	13–18 y	−0.4 (−1.5 to 0.8)	NS	
	19–30 y	1.0 (−0.02 to 2.4)	NS	
	≥31 y	0.04 (−0.7 to 0.8)	NS	
	Ayr	0	–	
	Maliboda	−1.3 (−2.1 to −0.6)	<0.0005	
	Interactions:			
	Co-infection: 13–18 y	−0.7 (−2.0 to 0.7)	NS	
	Co-infection: 19–30 y	−2.0 (−3.4 to −0.8)	<0.005	
	Co-infection: ≥31 y	−0.5 (−1.4 to 0.3)	NS	
	Co-infection: plantation	1.0 (0.1 to 1.9)	<0.05	

CI: confidence interval; NS: not significant, p>0.05.

**Table 3.** Negative binomial generalized linear models assessing the relationship between faeces egg count and the presence of co-infecting species and other factors, with all model variables, regression coefficients and variance-based R^2^

Species	Variable	Coefficient (eggs/54.5 mg) (95% CI)	p-Value	Variance-based R^2^ (%)
*Ascaris*	*N. americanus*	−0.3 (−0.7 to 0.06)	NS	1.4
	*Trichuris*	0.7 (0.2 to 1.1)	<0.005	
*N. americanus*	*Ascaris*	0.4 (0.0 to 0.8)	NS	1.4
	*Trichuris*	1.0 (0.4 to 1.6)	<0.0005	
*Trichuris*	*Ascaris*	0.05 (−0.3 to 0.4)	NS	0.3
	*N. americanus*	0.6 (0.06 to 1.2)	<0.05	
	Ayr	0	–	
	Maliboda	0.5 (−0.1 to 1.2)	NS	
	Water from waterfall	0	–	
	Pipe-borne water	−0.1 (−0.8 to 0.4)	NS	
	River water	0.1 (−1.0 to 1.6)	NS	
	Well water	0.7 (−0.2 to 1.6)	NS	
	Interaction:			
	*N. americanus*: Plantation	−1.0 (−1.7 to −0.4)	<0.005	

CI: confidence interval; NS: not significant, p>0.05.

Although there were significant effects in these models, they explained very little of the variation in the data, as demonstrated by the low R^2^ values (Tables 2–3). *Trichuris* deposition models had the lowest R^2^, at 0.3%.

## Discussion

We analysed data on STH egg deposition from two plantation communities in Sri Lanka for associations between STH species in infection prevalence and egg depositions.

### Associations between worm species

We analysed the associations between worm species and found strong positive associations between *N. americanus* and *Trichuris*, and *Ascaris* and *Trichuris* (Table 1, Figure 1). *Ascaris* and *Trichuris* share a faecal–oral transmission route, and we would expect similar exposures to correlate with the prevalence of both, leading to a positive association between them. *N. americanus* is transmitted differently, mainly through contact with bare skin, so it is unclear how the positive association between *N. americanus* and *Trichuris* arises. It is possible that socio-economic factors or pre-disposition, not captured in these models, correlate with *N. americanus* as well as *Trichuris*, although we might then expect there to also be a correlation with *Ascaris*. If within-host interactions lead to a benefit for establishment of infection of second and third species, we might also expect a positive association. These results may then indicate that establishment interactions differ between species, as there is no positive association between *N. americanus* and *Ascaris*, but positive associations in other cases. Figure 1 indicates that the proportion of the population that is uninfected or triply infected is greater than would be expected by chance. This could support the theory that establishment of second and third species is facilitated compared with establishment of primary infection. However, it is also important to consider that those with higher exposure may have stronger immune protection from worm establishment and stronger anti-fecundity immune responses.^26,27^ This may lead to cancelling out of positive associations due to correlated exposures. In addition, the amount of variation in the data explained by these models was low, suggesting that if there are correlated exposures, predispositions or establishment interactions, they are not strong drivers of STH infection.

Other studies in human and animal populations, including a large pooled analysis from Howard et al.,^6^ have tended to find positive associations between STH species infections, suggesting there is a higher prevalence of STH co-infections than would be expected by chance.^7,9,12^ These associations have been attributed to synergistic interactions within the host,^7,12^ correlated exposures^9^ and host predisposition.^6^ Comparing the results of these studies with the analysis presented here suggests that many factors are important in determining the distribution of STH co-infections, including STH species-specific within-host interactions, host-related factors such as human behaviour and other environmental factors.

### Egg deposition

There was a varied relationship between co-infection and egg deposition among STH species in this study. When assessing the difference in egg deposition between single infections and co-infections, both *N. americanus* and *Ascaris* egg deposition was higher in co-infections than in single infections, but this was not apparent in *Trichuris*. We found evidence of increased egg deposition in *Ascaris* and *N. americanus* infections when *Trichuris* was present, but no clear evidence of a similar effect in *Trichuris*. All egg deposition models had a low R^2^, highlighting that even though co-infection was found to have an effect in some models, this effect is fairly small.

Egg deposition is influenced by both adult worm number and per-worm fecundity—that is, greater egg deposition could indicate higher worm burden in co-infections, increased fecundity of a similar number of adult worms or both. By considering egg deposition findings alongside evidence of positive associations between species, we can suggest which of these may be the case. Increased egg deposition in co-infections coupled with a positive association in prevalence could indicate a benefit to establishment of second or third infections, leading to greater numbers of co-infections and greater worm burdens in co-infections. Increased egg deposition with no positive association, on the other hand, could indicate that the presence of co-infections improves the fecundity of a helminth but not establishment. We can therefore suggest that these results indicate positive effects of *Trichuris* on the establishment of *N. americanus* and *Ascaris*, leading to greater egg depositions and positive associations in each. *Trichuris* deposition is not affected by co-infection, however, suggesting that it is not facilitated by co-infections.

However, this analysis has limitations and there are alternative theories that should also be considered. To confirm these theories, data from different populations and larger samples are needed. For example, a possible alternative explanation for the higher egg deposition rate in co-infections could be that there is host predisposition to both higher-intensity infections and to multiple infections rather than there being interactions between species. It is also important to note that equal levels of egg deposition in co-infections to single infections could be due to no change in either establishment or fecundity or to changes in both that cancel each other out. Worm expulsion studies could give a better indication of the effect of co-infection on the number of adult worms.

Although several previous studies have found evidence for co-immune modulation in co-infections of STH, evidence for changes in the intensity of infection or egg deposition is mixed.^7,9,10,14^ We might expect co-immunoregulation to have some impact on the number of adult worms and adult worm fecundity, but this relationship is difficult to study in human populations, where other factors such as age and the presence of other non-STH infections such as malaria and *Schistosoma* will also interact with worm life history.

Overall, both our study and previous studies highlight that STH life cycles are complex and are not easily studied with indirect measures such as egg deposition. It is important to note that greater worm load could lead to greater morbidity and greater egg deposition could lead to greater transmission of parasites. Information about impacts to morbidity and/or transmission could inform STH treatment and control, so further investigation of this phenomenon is important, particularly as there is some evidence that infection intensity can affect drug efficacy and drug efficacy varies between STH species.^28–30^

### Limitations

The occupation and living conditions of the study population were relatively homogeneous, so these results have limited generalizability to other populations. Other studies of STH epidemiology take the prevalence of other non-STH infections into account, as there is evidence that there are also within-host interactions with these species. We do not have data on the prevalence of other infections in these communities at the time of this survey, so we could not analyse their influence on these data. The faecal sample used for the Kato–Katz microscopic assessment was less than a 1 g (54.5 mg); when this small, its representation of the egg concentration is sensitive to homogenisation of the sample. However, Kato–Katz is the recommended method for assessment of STH egg counts by the World Health Organization.^31^

## Conclusions

We found positive associations between some, but not all, STH species and some evidence that in *N. americanus* and *Ascaris* co-infection, egg deposition may be increased. The results may be due to correlated exposures, host pre-disposition or between-species interactions, but the variation between STH species suggests differential impacts of these factors on the different species. The within-host interactions are clearly complex, species specific and dependent on many other host- and environment-related factors. More needs to be known about the effects of co-infection on within-host life cycles of STH species to understand the importance of co-infection for morbidity and the epidemiology of co-infection.
